# The diagnostic value of ultrasound in cystic kidney diseases

**DOI:** 10.1007/s00467-008-0981-0

**Published:** 2010-02-01

**Authors:** Udo Vester, Birgitta Kranz, Peter F. Hoyer

**Affiliations:** Children’s Hospital, University of Duisburg-Essen, Hufelandstr. 55, 45122 Essen, Germany

**Keywords:** Renal cysts, Cystic kidney disease, Ultrasound, Children, Diagnosis

## Abstract

Renal cysts in childhood can be found in a variety of diseases, which can be congenital or acquired, or renal cysts may be part of a multiorgan disease or restricted to the kidneys only. Ultrasonography is the first-line diagnostic tool and is informative in many cases. However, there is a broad spectrum in the sonographic appearance of renal cysts, and family or genetic studies, a search for extrarenal organ involvement, or additional imaging modalities may be required to make a definitive diagnosis. The aim of this article is to summarize the diagnostic potential and limitations of ultrasonography and depict typical examples of the most important cystic entities.

## Introduction

Cysts are defined as spherical, fluid-filled, thin-walled structures that may be single or multiple. With the widespread availability of ultrasound, renal cysts in children can be diagnosed during the mother’s pregnancy or early childhood. There is no universally accepted classification for renal cysts, and according to a recent textbook: “it appears likely that cystic diseases of the kidney will be repeatedly reclassified with future insights into their pathogenesis” [1]. We have followed a proposal to classify developmental disorders of the kidneys to different stages of nephrogenesis [2], as follows:.

Renal cystic diseases classified to the time they develop in relation to the stage of nephrogenesis

1.
  Multicystic dysplasia
2.
  Dysplastic kidney with cysts
    Isolated
    As part of syndromes
    With obstruction
3.
  Systemic cystic renal diseases
    Autosomal recessive
    Autosomal dominant
    Nephronophthisis
    Medullary cystic disease
4.
  Isolated cysts
  Acquired renal cysts
  Cysts within tumors
  Metabolic diseases

It is the aim of this paper to summarize the technical requirements and sonographic images of renal cystic diseases and give examples of entities that should be familiar to the pediatric nephrologist.

## Technical requirements

Ultrasound fulfills the requirements of an ideal diagnostic tool in childhood: it does not expose the patient to radiation or contrast media, it can be repeated easily, it does not need patient preconditioning, and it offers good sensitivity and specificity. In addition, ultrasound allows family screening when indicated, with the same advantages. However, standardized and continuous operator training is essential, as it may reduce the “operator dependency” of ultrasound.

The kidneys can be visualized from both sides in a supine position. In older patients, the examination is mostly completed from the back with the patient in the prone position. Because of the well-defined interface to the surrounding tissue, ultrasound identification of cysts allows their visualization down to a size of 1 mm.

Modern ultrasound equipment should include probes suitable to depict the spectrum of renal diseases through the whole pediatric age range. This requires a sector probe and a linear probe of at least 8 MHz for infants and a 4-MHz sector probe for adolescents. Cysts in the kidneys are usually identified with the B-mode scan only, but modern technical facilities such as Doppler or harmonic mode may allow easier orientation or increase sensitivity.

It is important to emphasize that ultrasound in children with renal cysts should not be restricted to the kidneys, because multiorgan involvement in systemic cystic disease or syndromes should always be anticipated and included in the diagnostic workup.

Other imaging modalities may add additional information, such as magnetic resonance imaging (MRI) in extrarenal organ involvement or within interventional studies [3, 4]; radiological studies may help depict urological pathologies, such as vesicoureteral reflux; and scintigraphy helps measure renal function [5]. The growing knowledge of the genetic basis of cystic kidney diseases allows identification of hereditary origin [6].

## Renal cystic diseases

**Fig. 1:**
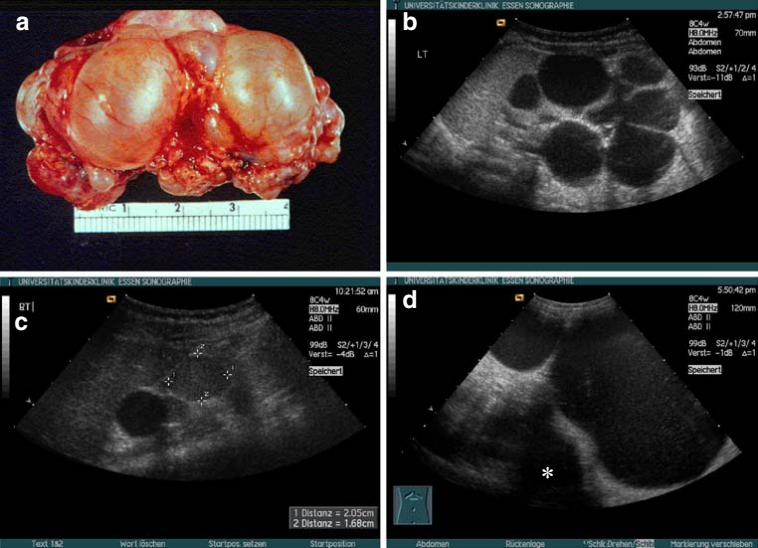
Multicystic dysplasia: **a** macroscopic appearance; **b** typical example of sonographic appearance; **c** multicystic kidney with nephroblastoma (*marked with calipers*); **d** giant-size multicystic kidney (approximately 900-ml volume) in a 2-year-old girl, crossing the midline (transverse section, * vertebral column)

*Multicystic renal dysplasia*. Multicystic renal dysplasia or multicystic dysplastic kidney is the most common cystic malformation of the kidney among infants. It is found in approximately 1 in 4,000 live births [7]. On ultrasound, the multicystic kidney typically consists of several variable-sized cysts without identifiable renal parenchyma between these cysts (Fig. 1a,b). The ureter is atretic in most cases, and the multicystic kidney does not show residual function. Bilateral disease is fatal in the newborn period, but usually only one kidney is affected. Most cases of multicystic renal dysplasia are suspected during prenatal ultrasound examination and should be followed after birth. Ultrasound often allows differentiation from severe hydronephrosis. However, in doubtful cases, scintigraphy to detect residual function or drainage may be indicated [5]. As involution of the multicystic dysplastic kidney is frequently observed [8], which can be followed sonographically, routine nephrectomy is not recommended and is reserved for some cases with hypertension or malignant transformation (Fig. 1c), or cases with an exceptionally large cystic kidney (Fig. 1d). As even after sonographic involution a remnant of the multicystic kidney can be expected, long-term follow-up seems advisable [9].

The contralateral kidney should receive special attention, as this kidney should hypertrophy to guarantee normal renal function and has been shown to have an increased incidence (20–40%) of minor malformations, such as vesicoureteral reflux [10–12]. Usually, multicystic dysplastic kidney is an incidental finding, but familial occurrence has been described [13].

*Renal dysplasia with cysts.* Renal dysplasia is a histological entity with undifferentiated parenchyma with or without cysts. Renal histology is characterized by poorly differentiated glomeruli, atypical tubuli, or nonrenal-tissue-like cartilage [14]. Renal dysplasia can affect one or both kidneys or may be segmental in some cases. Renal function of the affected kidney is more or less reduced; in bilateral cases, progressive loss of function may lead to renal failure. In large series, renal dysplasia is one of the leading causes of end-stage renal failure in childhood [15]. Therefore, monitoring renal function is more important than repeated ultrasound examinations.

Renal dysplasia with cysts may occur sporadically or as part of a variety of syndromes. A recent textbook of pediatric nephrology lists 78 syndromes, many of which can be suspected after recognition of associated malformations [16]. Therefore, siblings of some affected children may need to be screened to exclude familial forms of renal dysplasia.

**Fig. 2:**
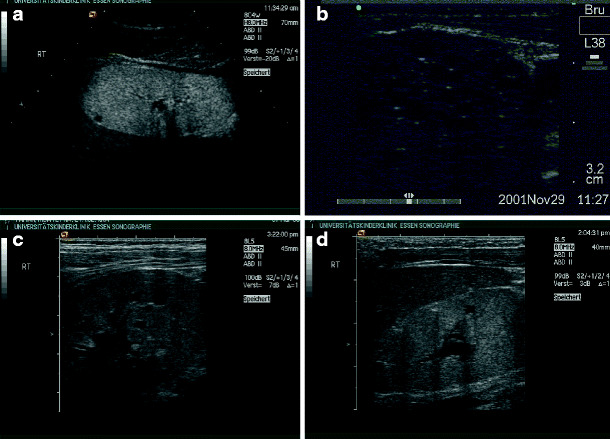
Renal dysplasia with cysts: **a** dysplastic kidney with a single and small cyst (13-year-old boy, creatinine 2.0 mg/dl); **b** dysplastic kidney with numerous cysts (2-week-old boy, end-stage renal failure within first year of life); **c**, **d** dysplastic kidneys with cysts and different function (**c** 6-year-old patient, creatinine 0.64 mg/dl; **d** 5-week-old patient, creatinine 2.0 mg/dl, end-stage renal failure at 6 months)

A typical sonographic feature of renal dysplasia is the lack of normal renal architecture, especially the differentiation between cortex and medulla. Echogenicity of a dysplastic kidney is usually enhanced, and cysts may be rare or numerous (Fig. 2a,b). Sonographic appearance does not necessarily correlate with renal function (Fig. 2c,d).

**Fig. 3:**
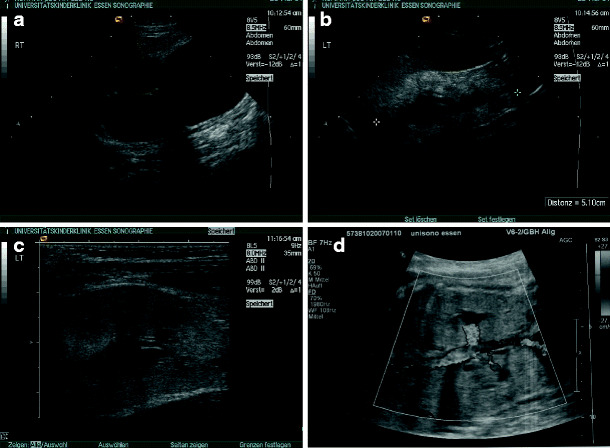
Phenotypic variability of renal dysplasia and cysts in a family with three siblings and hepatocyte nuclear factor (HNF)1β-mutation (mother has renal cysts, creatinine of 1.5 mg/dl and uterus bicornis): **a**, **b** index case (boy, end-stage renal failure within 10 months) (**a** multicystic dysplasia on the right side; **b** dysplasia without cysts on the left side); **c** sister, creatinine 1.0. mg/dl at the age of 1 year, dysplasia with cysts; **d** intrauterine enlarged kidneys in a third child, resembling autosomal recessive polycystic kidney disease (ARPKD)

It should be stressed that a distinct phenotype cannot be expected regularly within the same syndrome, as shown in Fig. 3a–d. In these three siblings with a mutation of the hepatocyte nuclear factor (HNF)-1β encoded by the *TCF2*-gene are shown. The phenotypic differences are obvious between right and left kidney in one patient (Fig. 3a,b) and between the siblings (Fig. 3c,d). HNF-1β mutations are found in up to 30% of unselected children with renal dysplasia and are frequently associated with maturity-onset diabetes of the young (MODY) type 5 and urogenital abnormalities [17].

**Fig. 4:**
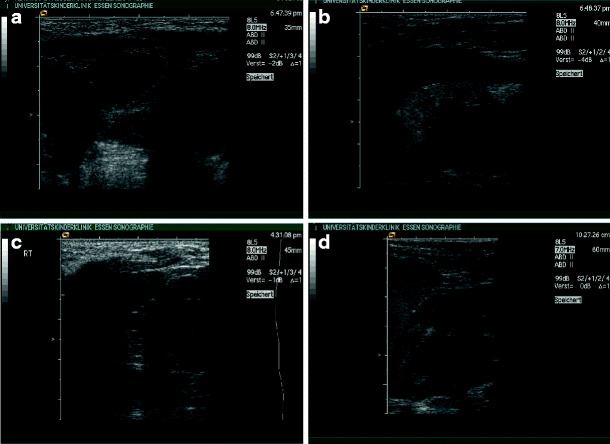
Renal dysplasia with cysts and urinary tract dilation (*CAKUT* congenital anomalies of kidney and urinary tract): **a**, **b** boy with urethral valves and impaired renal function (creatinine 1.5 mg/dl) at the age of 6 weeks (**a** dilated ureters and thickened bladder wall,** b** dysplastic right kidney with small cysts); **c** girl with duplex collecting system: the small upper kidney pole shows cystic dysplasia and the lower pole normal parenchyma; **d** upper kidney pole in a duplex collecting system, with lack of corticomedullary differentiation in the upper pole, indicating dysplasia without cysts

Renal dysplasia with or without cysts in combination with urinary tract abnormalities is called CAKUT (congenital anomalies of kidney and urinary tract) [18–20]. In Fig. 4a,b, a dysplastic kidney in combination with urethral valves is shown. Figure 4c, d depicts two children with duplication of the ureter and ureterocele with dysplastic parenchyma of the upper renal pole, in Fig. 4c with and in Fig. 4d without cysts.

**Fig. 5:**
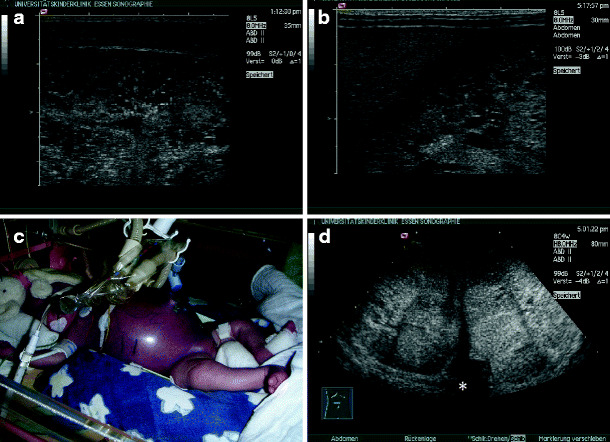
Autosomal recessive polycystic kidney disease (ARPKD) in neonates: **a** 2-week-old boy with typical echogenic spots representing small cysts; **b** 2-week-old girl with subcapsular brush-like appearance of dilated collecting ducts; **c** girl with giant-sized kidneys and ARPKD; **d** same patient as in** c**: transverse abdominal section with enlarged kidneys touching in the midline (* vertebral column)

*Autosomal recessive polycystic kidney disease (ARPKD)*. Autosomal recessive polycystic kidney disease (ARPKD) is caused by mutations in the *PKHD1*-gene on chromosome 6 [21–23]. The clinical spectrum of ARPKD ranges from intrauterine death to early renal failure and hypertension or preserved renal function into adulthood [24]. In pre- and postnatal ultrasound, the kidneys appear grossly enlarged, with increased echogenicity and reduced corticomedullary differentiation [25, 26] (Fig. 5a–d). Renal cysts are confined to the collecting ducts and are usually so small that their size in infancy is close to the resolution capacity of ultrasound.

**Fig. 6:**
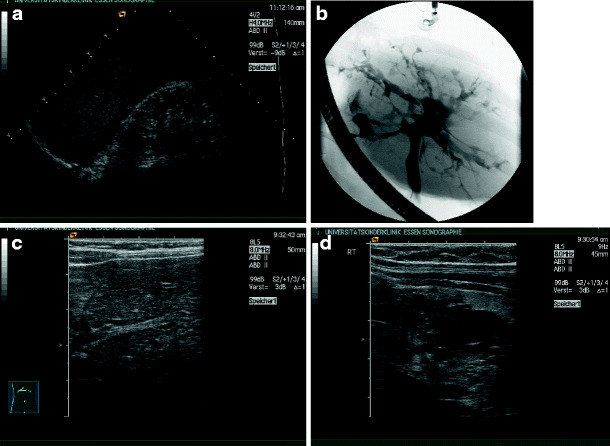
Autosomal recessive polycystic kidney disease (ARPKD) in advanced cases: **a** 8-year-old boy with peritoneal dialysis and portal hypertension – left longitudinal view with an enlarged spleen, free abdominal dialysis fluid, and polycystic left kidney; **b** cholangiodysplasia in the same patient (later received successful combined kidney–liver transplantation); **c** 9-year-old girl with thickened periportal echogenicity as a sign of periportal fibrosis (transverse liver scan); **d** numerous macroscopic cysts in the kidney of the same girl (cysts are much larger than in neonates with ARPKD)

In advanced cases, renal cysts may become bigger, and progressive hepatic fibrosis will lead to portal hypertension with splenomegaly (Fig. 6a–d). Follow-up ultrasound is requested mainly to monitor hepatic fibrosis and portal hypertension.

The variability of organ involvement in ARPKD is only partially understood [27] but is in part caused by the combination of mutations in the fibrocystin gene [28].

**Fig. 7:**
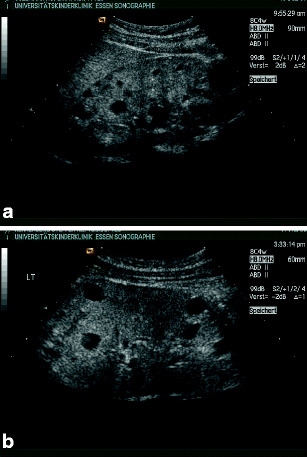
Typical sonographic image of autosomal dominant polycystic kidney disease (ADPKD): **a** 10-year-old girl (father ADPKD); **b** 11-year-old boy (father ADPKD)

*Autosomal dominant polycystic kidney disease (ADPKD)*. Autosomal dominant polycystic kidney disease (ADPKD) is the most common form of cystic disease, with a frequency of 1 in 800 live births and is caused by mutations in the *PKD1* gene on chromosome 16 or the *PKD2* gene on chromosome 4 [21]. With prenatal ultrasound, increased echogenicity of the renal cortex with increased corticomedullary differentiation is often found, but these findings are not specific [25]. In these cases, a family study is helpful, albeit most cases in childhood will be examined due to a positive family history. Clinical symptoms of ADPKD, as with hypertension and progressive enlargement of the kidneys and renal failure, are mainly seen in adult patients and are more severe in *PKD1*-related cases [3]. In childhood, cyst formation can be detected in increasing number and size, which develop in an apparently normal kidney (Fig. 7a,b). However, cyst formation is a developing process, and a significant number of children with ADPKD will not show cysts before the second decade of life [29]. Renal failure is exclusively seen in individuals with severe kidney enlargement [30].

In some cases of ADPKD, tuberous sclerosis cannot be distinguished by kidney morphology [31]. This has been identified as a contiguous gene on chromosome 16 [32]. These cases have to be diagnosed with additional investigations on extrarenal manifestation, such as brain MRI, to detect cerebral hamartomas [5].

**Fig. 8:**
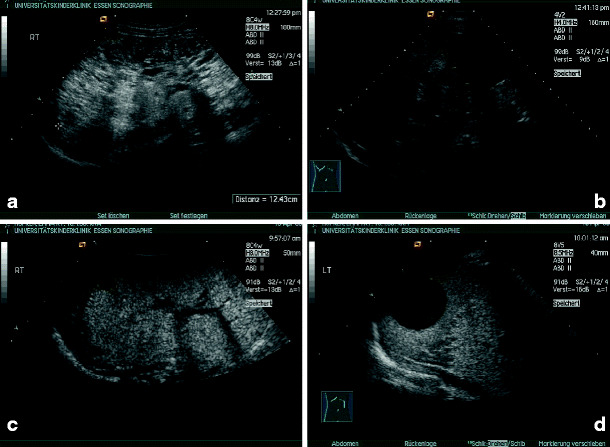
Autosomal dominant polycystic kidney disease (ADPKD) resembling autosomal recessive polycystic kidney disease (ARPKD): **a** 3-year-old boy with enlarged kidneys and small cysts; **b** father (38 years) of patient in** a** with multiple liver cysts and ADPKD; **c** 3-week-old girl, oligohydramnios, hypertension, small cysts in large kidneys, with a calculated total kidney volume of 90–100 ml (normal value < 40 ml according to [49] – mother ADPKD); **d** isolated spleen cyst in the same girl

A special subgroup of patients with ADPKD may exhibit symptoms early in life [33], and it can be sonographically confused with ARPKD (Fig. 8a–d). Outcome in this subgroup of children with ADPKD seems to be better than in children with ARPKD [34]. Classification in dominant or recessive cystic kidney disease therefore always requires family studies.

Extrarenal manifestation of ADPKD may involve mitral valve ballooning, cerebral aneurysm, or cyst formation of other parenchymal organs, such as the liver, spleen (Fig. 8b,d), or pancreas [35]. As in ARPKD, the variability of ADPKD is only partially understood, with genetic, environmental, and hormonal modifiers identified so far [27]. In families with recessive or dominant forms of polycystic kidney disease, siblings should be screened, albeit prenatal ultrasound is not always conclusive [36].

**Fig. 9:**
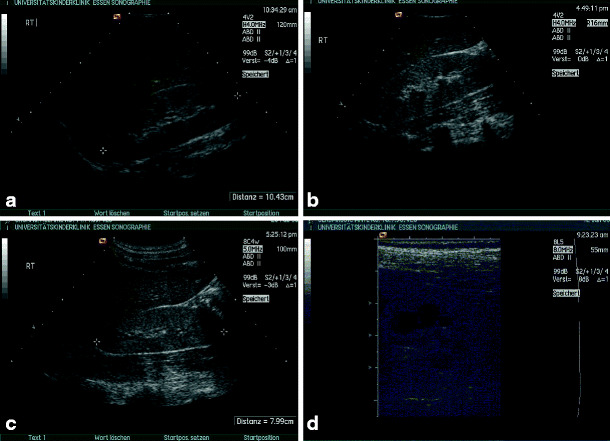
Juvenile nephronophthisis – sonographic appearance: normal or reduced kidney size, enhanced echogenicity of the renal cortex, and reduced corticomedullary differentiation (progressive with renal failure): **a** 13-year-old boy, creatinine 1.1 mg/dl; **b** 16-year-old boy (brother of patient in** a**), creatinine 2.4 mg/dl; **c** 16-year-old girl, end-stage renal failure; **d** 9-year-old boy, end-stage renal failure (cysts are a late sign)

*Nephronophthisis*. The nephronophthisis complex consists of several heterogeneous autosomal recessive diseases in which gene products are linked with cilia or centrosomes [37]. The sonographic appearance of nephronophthisis at an early stage is nonspecific, with normal-shaped kidneys [38]. A reduction in corticomedullary differentiation can be detected early. Albeit nephronophthisis is a cystic kidney disease, cysts are seldom found at the initial stage of disease and mostly do not appear before end-stage renal failure is reached (Figs. 9a–d).

Diagnosis of nephronophthisis with sonography alone is ambiguous, and the final diagnosis is made in combination with the typical clinical signs and extrarenal manifestations and is confirmed by genetic testing [39].

*Medullary cystic kidney disease (MCKD)*. MCKD is an autosomal dominant disorder with cystic dilation of the medullary part of the collecting ducts, which presents with gout and hyperuricemia in some cases and usually does not present before adulthood. So far, two loci on chromosomes 1 and 16 have been located. For the latter, mutations in the *UMOD* gene have been described [40]. Ultrasound examination is nonspecific, similar to nephronophthisis.

**Fig. 10:**
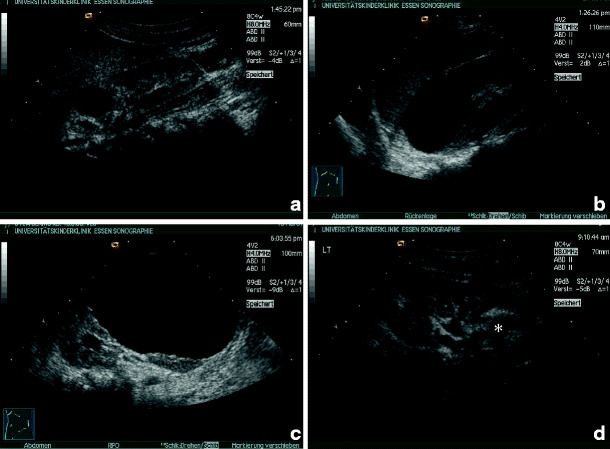
Isolated renal cysts with normal renal function and absence of familiar cystic kidney disease: **a** 3-month-old boy, isolated cyst of the right kidney – family members normal; **b** 15-year-old girl, incidental finding of an isolated cyst of the left kidney; **c** 3-year-old boy, isolated large cyst (size 10 × 9 × 6 cm , approximately 270 ml) **; d** same boy as in** c** after surgery (* shows former cyst)

*Isolated cysts*. Whereas benign cysts are found in up to 50% of the population older than 50 years, it is a rare finding in children [41]. Isolated cysts may be small or large (Fig. 10a–d). Multiple or bilateral cysts should always prompt suspicion to ADPKD and initiate family studies or follow-up examinations.

**Fig. 11:**
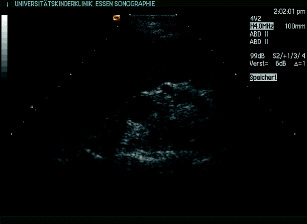
Acquired renal cysts in end-stage renal failure: 16-year-old girl with Alport syndrome and peritoneal dialysis from the age of 2 years

*Acquired cystic kidney disease*. Patients on dialysis often develop multiple cysts in their native kidney, even if the underlying disease is not cystic (Fig. 11). The pathogenesis of this cyst formation is not clearly understood, but the increased incidence of renal carcinomas in acquired cystic disease requires frequent and life-long monitoring of these kidneys [42, 43]. Regular follow-up and computed tomography (CT) or MRI in doubtful cases is advised, and removal of the nonfunctioning kidney should be considered in select cases [44].

**Fig. 12:**
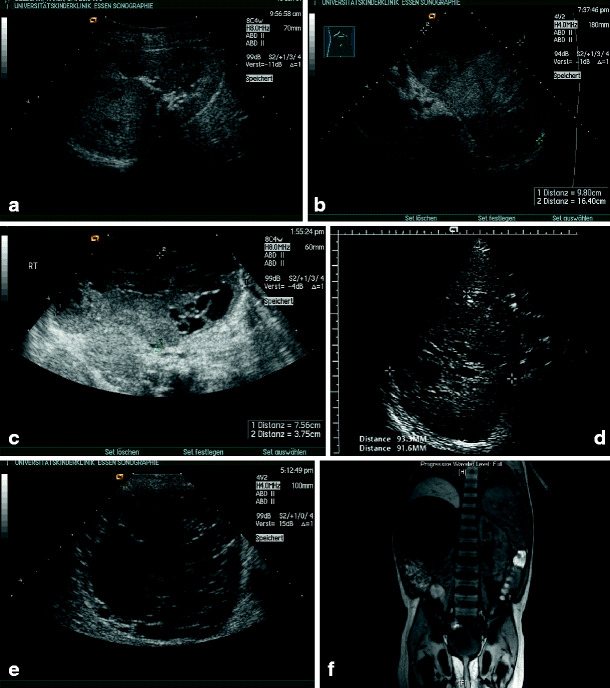
Cysts in renal tumors: **a** 9-year-old girl, small Wilms tumor with cysts; **b** 4-year-old boy, huge Wilms tumour with cysts of left kidney; **c** 2-week-old boy, mixed cystic and solid variant of a mesoblastic nephroma; **d** huge Wilms tumour with multiple cysts (transverse section); **e**, **f** 3-year-old boy, large kidney with multiple cysts – not a tumour but cystic dysplasia on histology; **e** transverse ultrasound, **f** magnetic resonance imaging

*Cysts within tumors*. It is of obvious importance to emphasize that tumors might harbor some degree of cystic parenchyma with variable expression [45]. This includes multilocular cystic nephroma and cystic variants of clear-cell sarcoma, renal cell carcinoma, nephroblastoma, or mesoblastic nephroma and should always be differentiated from benign lesions (Fig. 12a–f).

*Miscellaneous*. Cysts have been described in kidneys of patients with metabolic diseases such as glutaric acidemia type II or carnitine palmitoyltransferase type II deficiency or congenital disorders of glycosylation [46, 47].

## Summary

Ultrasound is convenient to use through the entire pediatric age range, as it does not need sedation or preconditioning and does not expose the child to radiation. Modern equipment allows the diagnosis of nearly all variants of cystic kidney disease. Therefore, ultrasound is the very first diagnostic tool and will guide further diagnostic workup with additional imaging studies or genetic testing. However, knowledge of the phenotypic variety of cystic renal diseases [48] is essential to correlate the sonographic appearance within the clinical and genetic context. Training in ultrasound, or at least knowledge of sonographic interpretation, is part of the training in pediatric nephrology, and we advise every trainee to concentrate on this rewarding technique for the benefit of their patients.
